# Trends in the incidence of cancers of the male genital system and urinary bladder in Harare, Zimbabwe, 1990–2019

**DOI:** 10.1007/s10552-025-02044-w

**Published:** 2025-08-16

**Authors:** Eric Chokunonga, Margaret Borok, Alex P. Danso, Mike Chirenje, Rudo Makunike-Mutasa, Ntokozo Ndlovu, Biying Liu, Justice Mudavanhu, Donald M. Parkin

**Affiliations:** 1 Zimbabwe National Cancer Registry, Parirenyatwa Group of Hospitals, Harare, Zimbabwe; 2 Department of Internal Medicine, Faculty of Medicine and Health Sciences, University of Zimbabwe, Harare, Zimbabwe; 3 Pioneering Health Group, Harare, Zimbabwe; 4 Department of Obstetrics, Gynaecology and Reproductive Science, University of California San Francisco, San Francisco, USA; 5 Department of Laboratory, Diagnostic and Investigative Sciences, Faculty of Medicine and Health Sciences, University of Zimbabwe, Harare, Zimbabwe; 6 Department of Oncology, Medical Physics and Imaging Sciences, Faculty of Medicine and Health Sciences, University of Zimbabwe, Harare, Zimbabwe; 7 African Cancer Registry Network, Oxford, UK; 8 Department of NCDs, Ministry of Health and Child Care, Harare, Zimbabwe; 9 International Agency for Research on Cancer, Lyon, France; 10 Nuffield Department of Population Health, University of Oxford, Oxford, OX3 7LF UK

**Keywords:** Cancer, Trends, Incidence, Prostate, Penis, Bladder, Zimbabwe, Registry

## Abstract

**Purpose:**

The cancer registry of Harare, Zimbabwe, founded in 1986 allows the study of the evolution of the cancer epidemic in a Black (African) population over a 30-year period, and is used to investigate trends in the incidence of cancers of the male genital system and urinary tract.

**Methods:**

Age standardised incidence rates (ASRs) in the black (African) population of Harare are calculated for four cancers: prostate, testis and penis in men, and bladder in both sexes. Trends are expressed as the average annual percentage change in incidence.

**Results:**

The incidence of prostate cancer is very high (an ASR of 71.4 per 10^5^) and over the period increased at a rate of 5.1% annually, but even faster (6.1%) in the most recent decade. The incidence of penile cancer is high, and has increased significantly (3.8% per year), while there was no change in the incidence of testicular cancer. Bladder cancer has shown significant declines in incidence in both sexes (1.9% annually in males, 3.8% in females). There has been little change in the histological composition of the bladder cancer cases in the last 25 years, with transitional cell carcinomas comprising some 50–60% of cancers.

**Conclusion:**

While some of these trends are related to population-level changes in lifestyles, and exposure to environmental factors (such as HPV), the reasons for other changes in incidence are more obscure. Some may be in part due to improvements in diagnostic techniques (endoscopy and imaging), but others—e.g. bladder—merit further investigation.

**Supplementary Information:**

The online version contains supplementary material available at 10.1007/s10552-025-02044-w.

## Introduction

Very few cancer registries in Africa are able to document the evolution of cancer patterns over a substantial period of time. The Zimbabwe National Cancer Registry was founded in 1986, and achieved complete coverage of the population of the capital city of Harare in 1990 [1]. Incidence rates for this population have been published in six successive volumes (VII–XII) of “Cancer Incidence in Five Continents.” [2].

As in much of Africa, progressive urbanisation has resulted in social and lifestyle changes in the population in last 50 years, resulting in changes to the profile of environmental risk factors for cancer. This has been reflected in the profile of cancer, which has been described in a previous paper presenting cancer incidence data from the registry for a 20-year period (1991–2010) [3]. In this article, we update this analysis, including more recent data, focussing on cancers of genital tract of men (prostate, testis, and penis) as well as of the urinary tract (bladder) in both sexes, in the black population of Harare over a 30-year period, from 1990 to 2019. The cancer profile of the white population is very different [4], and although a small proportion of the total (0.8% of the Harare population in 2012), it has been ageing much faster than the black population, so that time trends for the entire population are difficult to interpret.

## Materials and methods

### Cancer registration

The methods employed by the Zimbabwe National Cancer Registry in Harare have been described previously [3, 5]. Briefly, the registry is situated in a major referral hospital for the northern part of the country (Parirenyatwa Group of Hospitals). It collects information on cancer patients diagnosed and treated in all hospitals and clinics, public and private, as well as all pathology laboratories in the city, both by voluntary notification from certain institutions, and by staff visits. The registry uses death registrations as an important source of information on cases that may have been missed by the registration process. Cases identified from death registrations are followed up to obtain additional information on the diagnosis and management of the cancer, and if this is unsuccessful, cases are registered based on the death certificate only DCO). The information collected on each case includes patient demographic data, as well as details of the tumour, its treatment, the source(s) of information, and follow up (date of last contact or death). Information is collected on abstract forms, which is coded and entered into the computer using the CanReg5 cancer registration software provided by the International Agency for Research on Cancer. Tumour site and morphology are coded according to the third edition of the International Classification of Diseases (ICD) for Oncology [6]. For tabulation of results, these were converted to the 10th revision of the ICD.10.

### Population

**Fig. 1:**
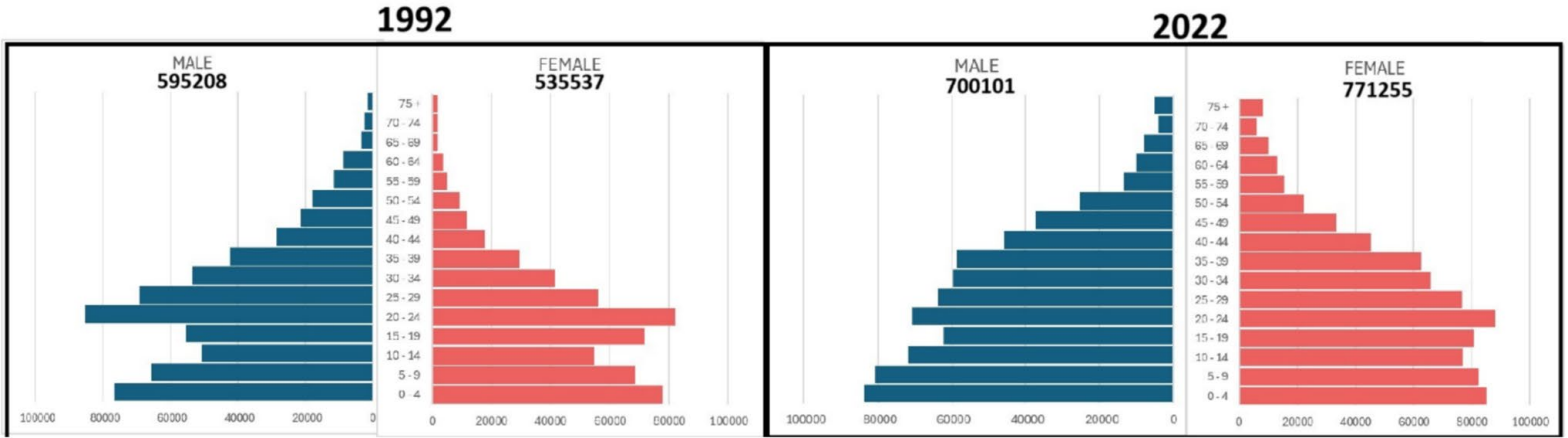
Population Pyramids for the black (African) population of Harare (1992 and 2022 census)

Population censuses were performed in 1992, 2002, 2012 and 2022, and for these years, the population of Harare was available by sex, ethnic group and 5-year age group from ZimStat (the Zimbabwe Statistics Agency). Annual intercensal estimates were prepared, assuming a constant rate of growth within age groups between census counts. Figure 1 shows population pyramids for the black population at the beginning (1992 census) and end (2022 census) of the period studied.

### Statistical methods

Incidence rates were calculated for the black population of Harare by 5-year age groups and sex, for each year (1990–2019), for six 5-year time periods: 1990–1994, 1995–1999, 2000–2004, 2005–2009, 2010–2014, 2015–2019, and for the entire 30 year period (1990–2019).

Age standardized rates (ASRs) were calculated using the World Standard population [7]. Temporal trends over the whole 30-year period were examined by fitting a regression line to the log-transformed age-standardized incidence rates. From this, we calculated the average annual percentage change (AAPC) as the slope of the regression line, together with its 95% confidence interval [8].

Two widely used indicators of data quality [9]—the percentage of cases with morphological verification (histology or cytology) of diagnosis (MV%) and the percentage of cases registered solely based on information on a death certificate only (DCO%) were calculated for each sex and the same periods.

Graphs of time trends in rates use 3-year moving average values of rates.

## Results

Table 1 shows age standardised incidence rates, as well as the number of cases, in six time periods, as well as the average annual percentage change (AAPC) of the ASRs over the whole period studied.

**Table 1:** Total number of cases registered, age standardised incidence rates (ASRs) in each of the 5-year periods, and the average annual percentage change (AAPC) in incidence over the period 1990–2019

	ICD O	Sex	Total cases	Age standardised rates (per 100,000)							AAPC (95% CICI)
				1990–1994	1995–1999	2000–2004	2005–2009	2010–2014	2015–2019	1990–2019	
Penis	C60	M	220	2.2	1.9	1.0	1.7	3.0	4.3	2.4	3.83 (0.32, 7.35)
Prostate	C61	M	4059	29.3	43.8	54.7	48.9	92.9	118.1	71.4	5.14 (4.20, 6.09)
Testis	C62	M	60	0.7	0.8	0.4	0.2	0.3	0.4	0.44	0.31 (− 3.55, 4.16)
Bladder	C67	M	560	9.0	8.3	7.9	7.3	6.2	5.5	7.3	− 1.93 (− 2.85, − 1.01)
		F	414	8.3	8.9	5.2	4.4	4.5	4.7	5.7	− 3.75 (− 5.30, − 2.19)

**Fig. 2:**
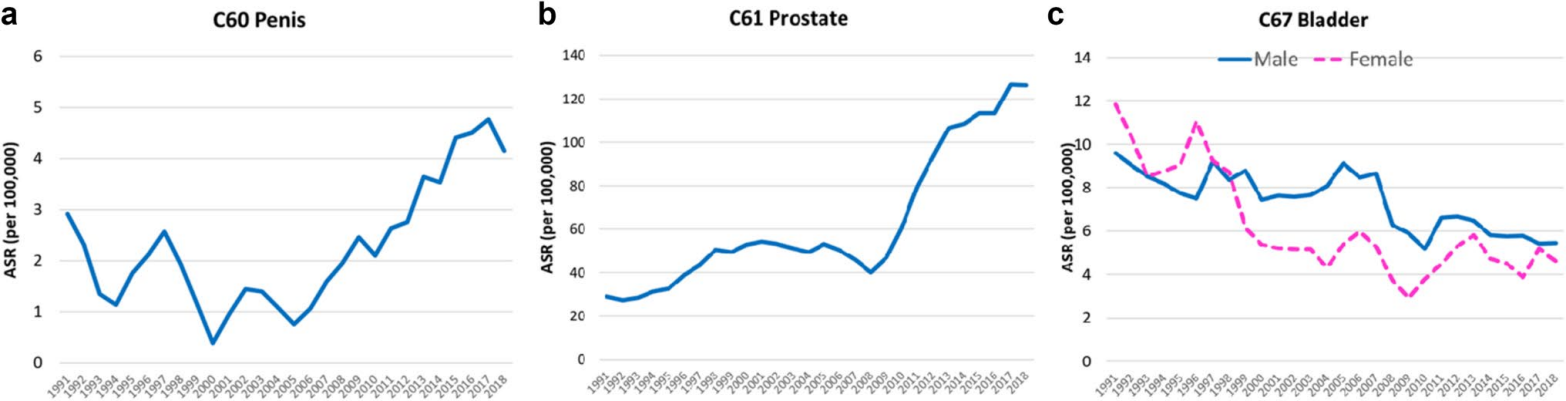
Trends in age standardised incidence rates (per 100,000) for cancers of the penis (**a**), prostate (**b**) and bladder (**c**). Values shown are 3-year moving averages

Figure 2 shows trends in the ASRs for cancers of the penis, prostate and bladder as 3-year moving averages.

We observe increasing incidence rates for cancers of the penis (AAPC 3.83 95% CI0.32, 7.35) and prostate (AAPC 5.14% 95% CI 4.20, 6.09) while the incidence of cancer of the bladder has been decreasing both in males (AAPC − 1.93% 95% CI − 2.85, − 1.01) and females (AAPC − 3.75%, 95% CI − 5.30, − 2.19).

For prostate cancer, it is clear that there is a change in the rate of increase after about 2008; with an increase in the most recent decade (2010–2019) of 6.1% annually (95% CI 3.1, 9.2).

**Fig. 3:**
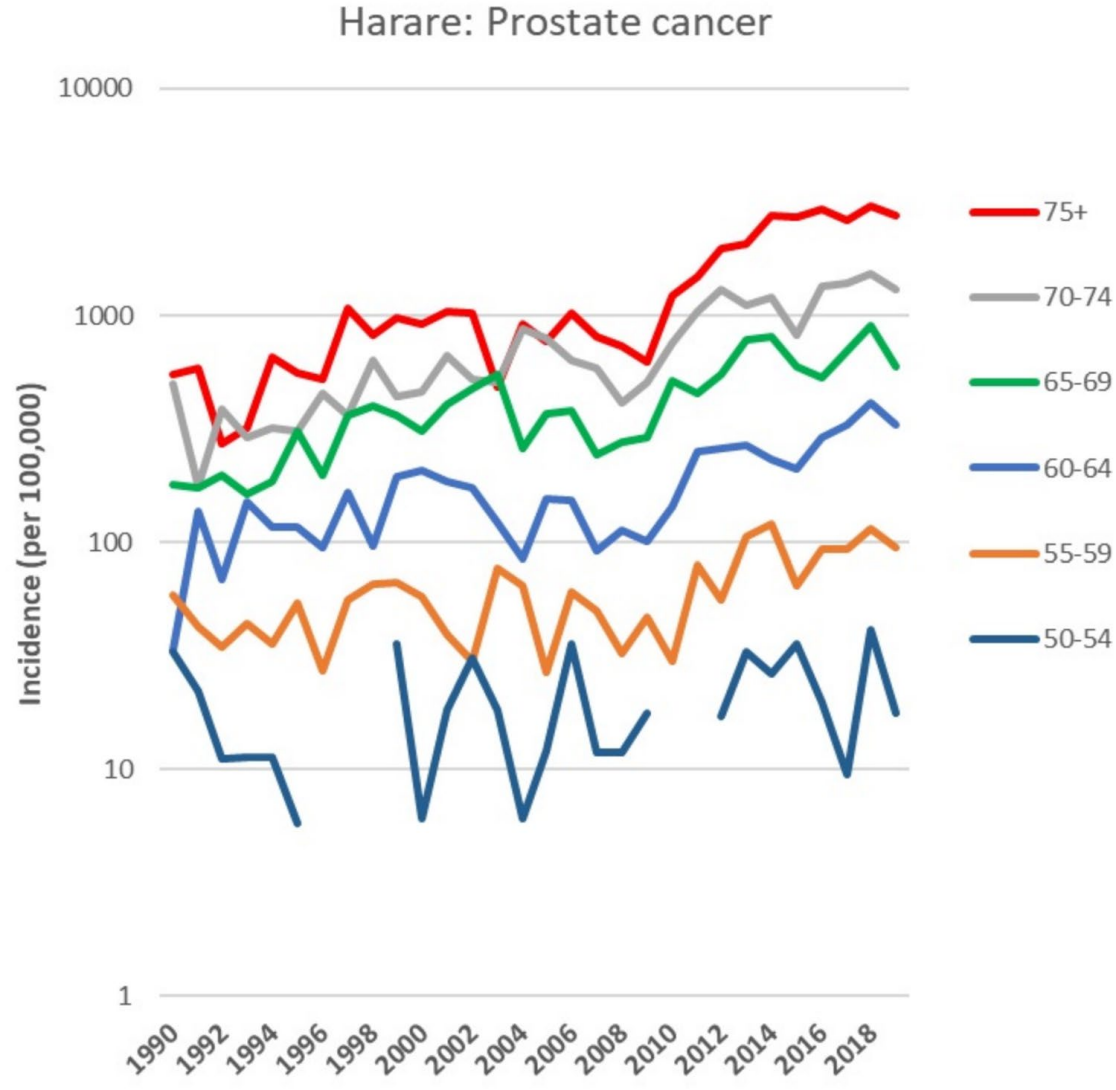
Trends in annual age specific incidence rates (per 100,000) for cancers of the prostate

Figure 3 depicts the age-specific incidence rates for prostate cancer.

**Fig. 4:**
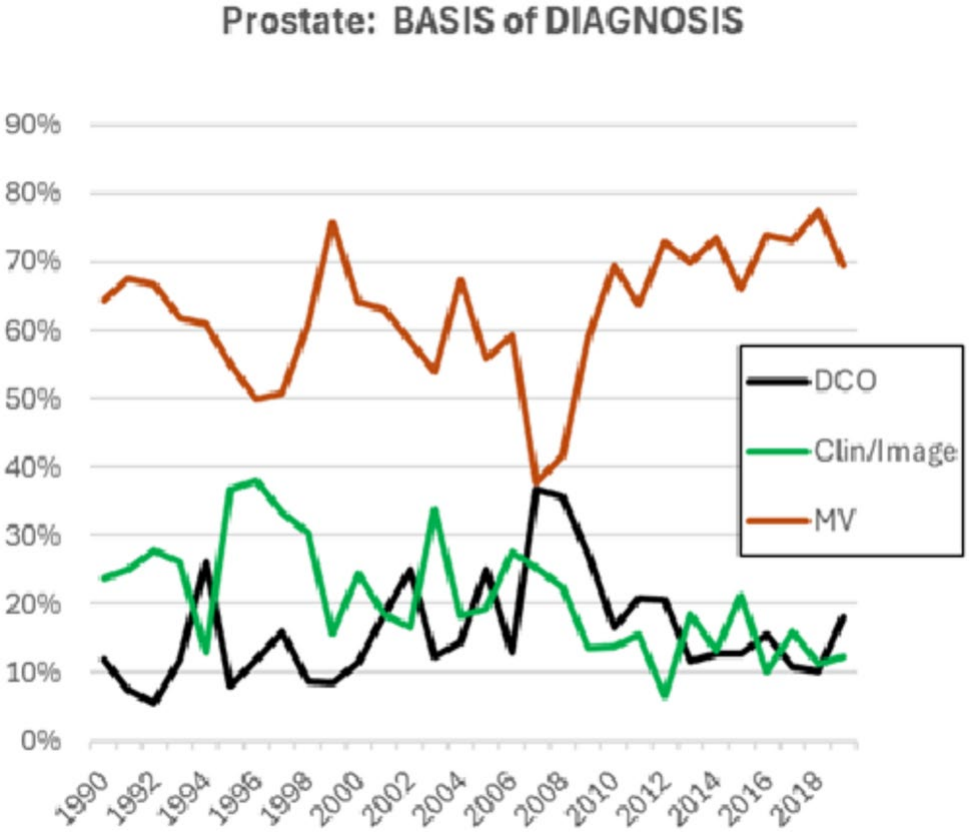
Trends in the annual percentage of cases of prostate cancer registered according to basis of diagnosis. *MV* morphological verification (histology or cytology), *Clin/Image* Clinical examinations and/or imaging, *DCO* Death certificate only

The brisk increase in rates after 2008 involves all age groups over the age of 55. Figure 4 shows the trends in the basis of diagnosis of prostate cancer cases (as percentages of registered cases). There is a small, but not very dramatic, increase in the proportion of cases with a morphological diagnosis (histology or cytology) in the most recent decade (72% in 2010–2019 compared with 61% in 1990–2006). The proportion of cases registered on the basis of information from a death certificate (DCO) has remained almost constant (at around 15%) over the entire period.

**Fig. 5:**
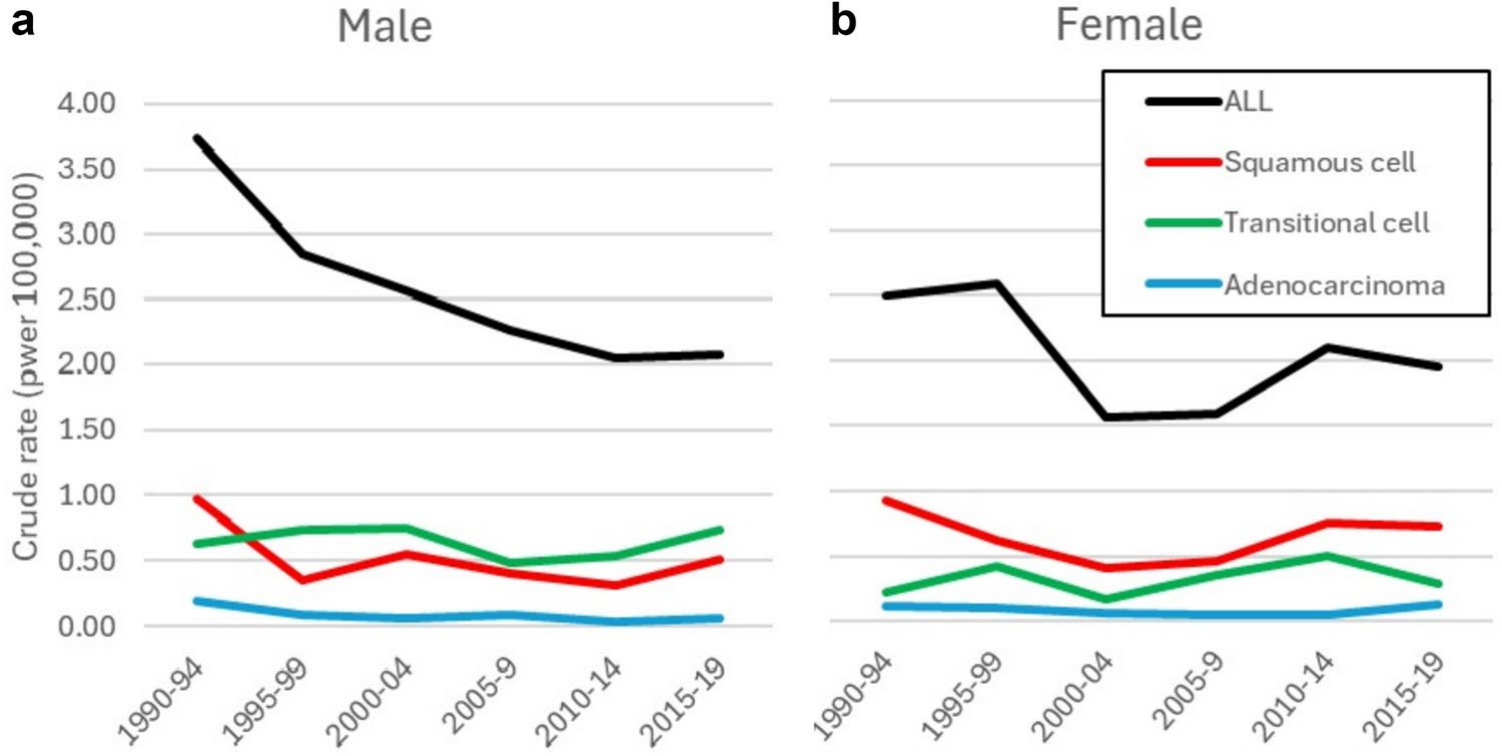
Trends in crude incidence rates (per 100,000) of bladder cancer by histological subtype, in six 5-year periods, 1990–2019. **a** Males **b** Females

For bladder cancers, the decline in incidence in over time is fairly constant. In males, 54% of cases had a morphological diagnosis, with a small improvement in the proportion over time (from 54% in 1990–2004 to 70% in 2015–2019), so that the proportion of cases of unknown or ill-defined histological type (“cancer”, or “carcinoma”) decreases over time. Similarly, in women, the percentage with a morphological diagnosis increased from 52% in 1990–2004 to 68% in 2015–2019. Figure 5 shows crude rates by histological subtype. In males, the incidence of squamous cell carcinomas decreased in the first 10 years, but after that, there has been little change in the relative proportions of subtypes, with transitional cell carcinomas making up the majority of cases with a defined morphology. In women, there appears to have been little change in the relative proportions of different histologies: squamous cell carcinomas comprise the majority of cases (with rates similar to those of males, while the incidence of transitional cell carcinomas is much lower (about half) than in males.

## Discussion and conclusions

Analyses of trends in incidence require that the degree of completeness of registration of cancer cases should be constant throughout the period under consideration. Results from the registry have been published in six successive volumes of Cancer Incidence in Five Continents from volumes VII (1990–1992) to volume XII (2013–2017) [2]. These volumes aim to present “….. high-quality statistics on cancer incidence from population-based cancer registries around the world.” [10]. The data quality indicators for Harare (MV% and DCO%) remain constant until 2005, but, as reported previously [4] there were significant political, economic and social problems in the years 2006–2009 that seriously impacted on the health sector, and the ability to diagnose (and hence register) cancer. In that earlier report on trends over 20 years, we omitted this period (the 3 years 2007–2009). However, these years are now towards the middle of the period studies, and although the decrease in registrations appears as a dip in the curve of incidence over, it does not much affect the overall trends shown in Table 1. We tested this by recalculating the AAPC, omitting the 3-year period 2007–2009 (Supplementary Table). Although this changed the actual values of the AAPC slightly, in no case was the statistical significance of the trend (from the null value) different, so we have retained the values incorporating the 3 years concerned.

The population of Harare is progressively increasing, from 1.1 million in 1992 to 1.5 million in 2022. There is substantial migration into the city—particularly of young adults seeking work—as can be seen from the shape of the population pyramids (Fig. 1). Along with this growth are changes in lifestyles, as the population becomes more urbanised, sedentary, with changes in diet away from home produce towards purchased items, with increased consumption of animal proteins and prevalence of overweight and obesity [11]. This demographic transition is accompanied by familiar trends in patterns of health and illness, with a decrease in maternal and infant mortality, and a rise in the importance on non-communicable diseases [12, 13]. It is therefore not surprising to note a steady increase in the overall incidence of cancer in both sexes, with the (age adjusted) incidence some 32% higher in 2015–2019 compared with 1990–1994.

The incidence of prostatic cancer in Harare is extremely high; in the most recent quinquennium (2015–2019) the age standardized rate was 118.4 per 100,000, far higher than any other population in Africa, and indeed, in the world; only black populations of African ancestry in the Americas have similar (or higher) incidence rates [10]. Incidence in Harare has been rising rapidly throughout the period, and especially fast (6.1% annually) since 2008.

A recent study of trends in twelve sub-Saharan African cancer registries showed that, although incidence rates varied up to sevenfold between populations, they had been increasing in all during the periods of observation [14]. This contrasts with observations of incidence rates in 44 countries worldwide (and mortality in 76) where prostate cancer rates were found to have stabilized in most, and decreased in a few, since 2008/2012 [15].

It has long been known that black populations of African origin (African-Americans, as well as Afro-Caribbeans) have one of the highest risks of being diagnosed with and dying from prostate cancer. [16, 17], but the reasons for the rising incidence in Africa have been little investigated. Environmental factors can certainly influence risk, as shown by migrant studies. These factors comprise, amongst others, metabolic syndrome, obesity, body size, and dietary factors. However, apart from obesity and body size, the evidence is still poor and controversial [18–20]. In addition, most studies are in populations of white European ancestry, and it is possible that the effects of these factors on risk in predominantly black populations might be different [21]. The prevalence of overweight and obesity is increasing in Zimbabwe: 6.8% of the adult population were obese in 2000, and this had risen to 13.2% in 2020 [11], but the risks associated with these putative risk factors are not large, and the huge increase in incidence can hardly be due to such changes.

The biggest influence on reported incidence of prostate cancer has been the introduction of early detection programs through PSA testing in asymptomatic men [22, 23], and recent declines in incidence in high income countries are ascribed to a reduction in such testing (or simply exhaustion of the pool of undetected asymptomatic cancers). Although there has been no formal widespread general PSA screening activity in Zimbabwe [24], increased awareness of the risk of prostate cancer, and PSA testing of symptomatic (or simply worried) men has most likely resulted in increasing detection of early, or asymptomatic cancers. This is consistent with the observation of an abrupt increase in incidence of histologically diagnosed cancers since 2008, reminiscent of the same phenomenon when PSA testing became widespread some 15–20 years earlier in the USA, Canada, Australia and Sweden [22, 23, 25, 26].

An ad hoc survey of AFCRN populations has confirmed that the PSA test is available in laboratories throughout Africa, and is widely used for diagnostic purposes [14], although there is no information on the trends in numbers of tests performed over time. It seems likely that at least some of the increase in incidence represents better detection (and diagnosis) of prostate cancers in middle-aged and elderly men with urinary symptoms. Increased diagnostic imaging with MRI and transrectal ultrasound scanning may also account for the increase in incidence of prostate cancer. The availability of MRI guided and transrectal ultrasound guided biopsies leads to a higher yield of diagnosis of prostate cancer as compared to blind transrectal biopsies which obtain in other parts of Africa.

Additionally, there might also be an increase in the availability and consequently in the performance of trans-urethral resections of the prostate (TURP) to treat urinary retention suspected to be caused by benign prostatic hyperplasia. The actual number of specialists performing TURP has tripled in Harare since 1992. This could account for more incidental carcinomas and rising incidence rates, as has been described by Potosky et al. for the USA in the pre-PSA screening era, during the years 1973 to 1986 [27].

The incidence of penile cancer in Harare – with an average ASR over the 30-year period of 2.4 per 10^5^—is relatively high, compared with the average in sub-Saharan Africa (0.7 per 10^5^) [28], although higher rates have been reported from Uganda and E-Swatini [5]. There has been an increase in incidence over the 30-year period, which was mainly due to rising rates in the most recent 20 years.

Some 40–50% of penile cancers are associated with infection with human papilloma viruses (HPV) [29]. Male circumcision in childhood protects against cancer of the penis [30], and circumcised men clear HPV infections more quickly [31]. The incidence of cancer of the cervix has been increasing in women in Harare [32], and although no data are available on the HPV burden in the general population of Zimbabwe [33], the most likely explanation is an increase in prevalence of HPV infection. Changes in family structures and social mores favour the spread of HPV (like other sexually transmitted diseases), resulting in increasing risk of cervical cancer, and, most likely, of penile cancers also. Although voluntary male circumcision was introduced in Zimbabwe in 2009, and, by 2016 an estimated 30% of young men (aged 15–49) had been circumcised, with a probable reduction in the transmission of HIV-AIDS in the population [34], this is unlikely to have had an effect on prevalence of infection with HPV in older men.

The incidence of bladder cancer is high in Harare—especially in women. The estimated average ASR for sub-Saharan Africa in 2022 was 3.6 per 10^5^ in men and 2.0 per 10^5^ in women [28]. Worldwide, diverging incidence trends were observed by sex in many countries, with stabilising or declining rates in men but some increasing trends seen for women [35]. The incidence of bladder cancer has declined markedly in Bulawayo in Western Zimbabwe since the 1960’s [36] and has been declining in Harare over the 30 years of this study.

Since tobacco smoking is the major risk factor for bladder cancer, the observed patterns and trends of bladder cancer incidence worldwide appear to reflect the prevalence of tobacco smoking. In Zimbabwe, smoking is much more prevalent in men (which may account for the higher rates, especially for transitional cell carcinomas), and there is a downwards trend in current smoking prevalence, decreasing from 33% in 2000 to 20% 2020 [37]. In women, current smoking prevalence was low at 3% in 2000, dropping slightly to 1% in 2020. These changes seem hardly sufficient to explain the trends observed, particularly the more rapid decline in risk in women.

In some regions, notably sub-Saharan Africa, infection with Schistosoma haematobium is an important risk factor, notably for squamous cell carcinomas [38]. Urinary schistosomiasis remains prevalent in Zimbabwe [39]; a national survey of schoolchildren in 2010–2011 suggested an average prevalence of infection of 23%, rather lower than this in urban Harare (9.6%) [40]. There is some evidence that prevalence is waning [41], which may also account for the observed declines in incidence; however there seems to have been little change in the proportions of squamous cell carcinomas, at least, since 1990’s.

## Conclusions

Studies of trends in incidence have proved valuable when considered in conjunction with the possible the reasons behind the observations and might help to enlighten important questions on the aetiology of the cancers concerned. Trends are also valuable in forecasting the likely future requirements for cancer control interventions. In Harare, as in almost all of sub- Saharan Africa, prostate cancer is the most frequent cancer of men and is rapidly increasing in incidence [14]. This surge in the numbers of cases cannot be prevented by lifestyle changes or public health interventions alone, and early diagnosis to improve prognosis and outcome (and reduce societal and individual costs) through changes to the diagnostic pathway that can be immediately implemented are a more logical approach to reducing mortality [42]. For bladder cancer, prevention measures involve continuing efforts to reduce tobacco smoking, and control of urinary schistosomiasis [43].

## Supplementary Information

Below is the link to the electronic supplementary material.

Supplementary file1 (DOCX 16 KB)

## Data Availability

The data that support the findings of our study are available on request to the Zimbabwe National Cancer Registry.
