# Supplementary material for: Trends in the incidence of cancers of the male genital system and urinary bladder in Harare, Zimbabwe, 1990–2019
Source: Cancer Causes Control. 2025 Aug 16;36(11):1579–87. doi: 10.1007/s10552-025-02044-w (PMC12578678; doi:10.1007/s10552-025-02044-w)
Supplement: Supplementary file 1 — Supplementary file1 (DOCX 16 KB) [file 10552_2025_2044_MOESM1_ESM.docx]

**Supplementary Material**

**TABLE S1**

Average annual percentage change (AAPC) in age standardised incidence using all data (1990-2019) and omitting years 2007-2009

| **MALES** | **ICD 10** | **AAPC (95% CI)**  **1990-2019** | **AAPC (95% CI)**  **Omitting 2007-2009** |
| --- | --- | --- | --- |
| **Penis** | C60 | 3.83 (0.32, 7.35) | 3.93 (0.46, 8.32) |
| **Prostate** | -C61 | 5.14 (4.20, 6.09) | 6.25 (5.48, 7.01) |
| **Testis** | C62 | 0.31 (-3.55, 4.16) | 0.67 (-3.80, 5.14) |
| **Bladder: Males** | C67 | -1.93 (-2.85, -1.01) | -2.07 (-3.01, -1.13) |
| **Bladder: Females** |  | -3.75 (-5.30, -2.19) | -4.16 (-5.71, -2.61) |
